# Sleep hygiene linked to patient-reported outcomes & objective sleep measures prior to upper extremity orthopaedic surgery

**DOI:** 10.3389/fpain.2025.1589748

**Published:** 2025-06-11

**Authors:** Nicholas A. Giordano, Tatiana Getz, Michael Gottschalk, Andrew H. Miller, Kim Dupree Jones, Jasmine Park, Yining Zhu, Annabelle Gong, Jack Hudson, Selma Selimovic, Sarah M. Taub, Amanda K. Klosky, Eric R. Wagner

**Affiliations:** ^1^ Nell Hodgson Woodruff School of Nursing, Emory University, Atlanta, Georgia; ^2^ Department of Orthopaedics, School of Medicine, Emory University, Atlanta, Georgia; ^3^ Department of Psychiatry and Behavioral Sciences, Emory University, Atlanta, Georgia

**Keywords:** actigraphy, orthopaedic surgery, pain, sleep hygiene, sleep quality

## Abstract

**Background:**

Sleep hygiene is a modifiable factor that influences sleep quality, which is vital to the body's healing process and pain response. However, poor sleep hygiene, characterized by irregular sleep schedules, inappropriate sleep environments, or the use of stimulants before bedtime, can exacerbate sleep disturbances and impairment, thus diminishing sleep quality, exacerbating pain hypersensitivity, and protracting postoperative recovery. Despite being modifiable, sleep hygiene is rarely assessed preoperatively and may be a driver of the relationship between poor sleep quality and pain response in surgical patient populations. Furthermore, there is a dearth of research examining the relationship between sleep hygiene and objective sleep measures in an ambulatory surgical patient population.

**Purpose:**

This analysis examined the association between sleep hygiene habits and both pain and sleep quality in a sample of patients undergoing orthopaedic surgery utilizing patient-reported outcomes and objective longitudinal measures of sleep quality, known as actigraphy.

**Methods:**

Participants undergoing orthopaedic surgery on their upper extremity at a large urban academic medical center in the Southeastern United States were recruited, consented, and enrolled in this study approximately 2 weeks before surgery between March 2022 and April 2023. Participants completed a series of surveys assessing their sleep hygiene, sleep quality, and pain interference preoperatively. For example, participants completed the Sleep Hygiene Index and the Patient-Reported Outcome Measurement Information System (PROMIS) Pain Interference measure. In addition, participants wore an actigraphy device on the wrist of their non-surgical extremity, which measured sleep efficiency and total sleep time in the days prior to surgery. Linear regressions examined the association between preoperative sleep hygiene scores and pain interference, sleep efficiency, and total sleep time.

**Results:**

This sample included 30 participants. The average Sleep Hygiene Index score was 10.87 (±6.71) and the sample's average PROMIS Pain Interference *T*-score was 63.73 (±9.59). Actigraphy derived total sleep time per day was 362.97 (±154.02) minutes and an average sleep efficiency score of 91.98 (±3.72). Regression models showed that poorer sleep hygiene (e.g., higher scores) was associated with worse PROMIS Pain Interference *T*-scores (95% CI: 0.14, 1.04; *p* = .04). In addition, participants with worse sleep hygiene scores had worse sleep efficiency (*β* = −0.21; 95% CI: −0.41, −0.014; *p* = .037) and had fewer minutes in their total sleep time (*β* = −8.91; 95% CI: −17.10, −0.72; *p* = .034).

**Conclusion:**

This analysis indicates that poorer sleep hygiene is associated with both increased pain interference and poorer sleep quality among patients about to undergo orthopaedic surgery on their upper extremity. These findings highlight the need to assess and educate patients on proper sleep hygiene prior to surgery in an effort to help foster high-quality restorative sleep that promotes postoperative recovery. This study is among the first to examine the possible contributions of sleep hygiene, a modifiable factor, on both patient-reported outcomes and objective measures of sleep over a prolonged period among patients undergoing orthopaedic surgery in an ambulatory setting.

## 1 Introduction

Most patients report poor preoperative sleep quality and frequent sleep disruption, which can significantly impact overall health and recovery after surgery. For example, estimates indicate that up to 60% of patients report poor quality sleep prior to surgery (1). Even higher rates of sleep impairments have been observed among patients undergoing orthopaedic shoulder surgery (2). Poor sleep quality and shortened sleep duration have been linked to worsening health outcomes among patients. Meta-analyses indicate that poor preoperative sleep is linked to increased odds of experiencing moderate to severe pain in the first days after surgery, heightened risk for delirium, and is associated with protracted recovery due to reductions in physical functioning (3–5). As such, there is a need to identify patients experiencing poor sleep preoperatively to better facilitate the timely implementation of interventions capable of bolstering sleep to ensure optimized postoperative recovery.

Sleep quality is vital to fostering postoperative healing, and sleep impairment prior to surgery may predispose patients to experience worsening outcomes. During sleep, particularly in the deep sleep stages, the body releases growth hormones for tissue repair and regeneration, facilitating healing of incisions and damaged tissues at the surgical site (6). Moreover, sufficient sleep enhances immune system function to reduce the risk of postoperative infections and complications (7). Sleep quality aids in pain management by diminishing pain perception and increasing pain tolerance, potentially lessening the need for pain medications that may have undesirable side effects (8). Furthermore, high-quality sleep is beneficial for mental health and well-being, having been found to reduce anxiety before surgery (8). The preponderance of research examining preoperative sleep quality depend upon patient-reported outcomes alone (3). Investigations incorporating objective measures of sleep architecture, such as actigraphy devices, with patient-reported outcomes among patients undergoing upper extremity orthopaedic surgery are warranted to better discern opportunities to improve sleep quality in the preoperative period.

Despite the importance of optimizing sleep quality to facilitate recovery in surgical patient populations, particularly in orthopaedic patient populations, clinicians rarely assess modifiable factors that can potentially improve sleep. Robust research indicates that poor preoperative sleep is associated with increased postoperative pain, increased analgesic medication use, reduced range of motion, limited physical functioning, lower satisfaction with care, and prolonged inpatient stays among patients undergoing orthopaedic surgery, yet limited research has examined modifiable factors to optimize sleep (1–3, 5). Rather most investigations focus on improving sleep quality focus on pharmacological approaches or postoperative (9–11). Poor sleep hygiene, such as irregular sleep schedules, unsuitable sleep environments, and the use of stimulants before bedtime, can disrupt the restorative processes by impairing patients' ability to stay asleep and achieve deep restorative sleep (12). Emerging evidence indicates the benefits of interventions aimed at improving sleep hygiene to improve subsequent sleep outcomes (13–15). However, less attention has been placed on identifying potential associations between sleep hygiene and both patient-reported and objective outcomes, which is needed in order to guide future intervention development and study designs. When screened preoperatively, sleep hygiene and sleep environments are modifiable factors that can be addressed to facilitate better sleep quality. Researchers have predominately focused on assessing sleep hygiene in hospitalized patient populations or in home settings after discharge (16, 17). Less research has examined sleep hygiene among patients preoperatively, a period when proactive interventions to promote sleep may be feasible to implement.

Studies ascertaining the sleep behaviors of patients prior to surgery can inform future research designing interventions aimed at fostering better sleep environments that help promote high quality sleep both before and after surgery. Doing so may help patients return to, if not improve upon, their preoperative sleep quality after surgery and, ultimately, optimize recovery outcomes. Therefore, this prospective study investigated the association between sleep hygiene and preoperative presentations—including patient-reported pain interference, objective sleep efficiency and total sleep time—among patients undergoing orthopaedic surgical procedures on their upper extremity. It was hypothesized that participants presenting with worse sleep hygiene would report worse preoperative outcomes.

## 2 Methods

### 2.1 Sample and setting

Patients undergoing orthopaedic procedures on their upper extremity at Emory University between March 2022 and April 2023 able to communicate in English were eligible to participate. Individuals under 18 years of age and those without a stable internet connected device were excluded. To mitigate confounding from non-surgical site related pain, individuals with a preoperative ICD-10 chronic pain diagnosis or self-reported widespread pain conditions (e.g., fibromyalgia, neuropathy, etc.), undergoing a revision procedure, with any allergy to opioid medications, unlikely to receive a prescription for an opioid, or with an active opioid prescription within a month before surgery were excluded. Additionally, individuals with sleep-related conditions (e.g., diagnoses of sleep apnea, insomnia, etc.) were excluded to ensure actigraphy based sleep data were accurately captured. In total, 329 patients were screened for eligibility and 44 patients met eligibility criteria, were consented by study staff, and enrolled in the study.

### 2.2 Procedures

Immediately following their preoperative consult with the orthopaedic surgeon, approximately 2 weeks prior to their surgery, the participants met with a study coordinator in private space to provide signed consent and complete survey measures. Participants were also provided a wrist worn actigraphy device to wear in the days leading up to their scheduled surgery. All participants received both physical and digital handouts on properly wearing actigraphy devices and were followed up with by the study coordinator via phone 3 days prior to surgery to confirm watch placement and address any questions from participants. Participants received $20 for completing the study. All data were securely entered onto Research Electronic Data Capture (REDCap) platform stored on University firewalled protected servers and deidentified for analysis. The Emory University Institutional Review Board (IRB) reviewed and approved this study (00003473).

### 2.3 Measures

Participants completed a series of patient-reported outcomes after consenting to be in the study that were captured electronically in REDCap. Additionally, data collected from actigraphy devices were securely uploaded, analyzed, and stored in REDCap.

Sleep Hygiene Index: The Sleep Hygiene Index consists of 13 items assessing overall sleep routine, environment, substance use, and napping habits (18). The index is scored based on a series of statements, with respondents indicating how often each behavior or habit occurs on a scale ranging from, 0, “never” to 4, “always”. Scores are summed to compute a total score on a scale from 0 (best) to 52 (worst) (19).

Patient-Reported Outcomes Measurement Information System (PROMIS) Pain Interference: The PROMIS Pain Interference short form measure includes 8 items assessing the degree to which pain interfered or impeded activities in the past week (20). Each item is scored on a scale from 1, “Not at all”, to 5, “Very much”. Scores are summed across all items to create a raw total score. This sum is then converted to a *T*-score, ranging between 0 and 100, where higher scores suggest greater pain interference, indicating that pain is significantly affecting the person's ability to function and carry out their regular activities (21).

Actigraphy Sleep Measures: Participants received and were trained on how to wear, a screen-less actigraphy device on their nonoperative side's wrist (GT3XP-BTLE, Actigraph, LLC). Devices were set to record in 30-s epochs at a medium sensitivity level for scoring sleep and wake time. Participants were instructed to return their devices to research staff during their scheduled clinical follow-up visit with the surgeon or asked to mail the devices back using a pre-paid envelope provided to them. Wear time validation was accomplished using the Choi algorithm (22). The sleep data were computed using the Cole-Kripke algorithm, which accurately distinguishes sleep from wakefulness 88% of the time (23). Total sleep time, computed in minutes, refers to the duration of time a participant spent asleep, as determined from the actigraphy movement and wear data. Sleep efficiency was calculated as the percentage of time spent asleep relative to the total amount of time spent in bed, reported as a percentage. Wakefulness after sleep onset (WASO) is the total minutes a person is awake after they have initially fallen asleep. The sleep fragmentation index represents a person's restlessness by using their movement and fragmentation, based on activity counts and fragmented sleep periods, to express this (24). A higher sleep fragmentation index is associated with a more restless sleep. Additionally, the number and length of awakenings are calculated per sleep period by the devices. These scoring practices and reporting measures align with recommended best practices for using actigraphy to examine health outcomes (25). Actigraphy devices were chosen over other sleep assessment approaches, such as polysomnography, due to its practicality, cost-effectiveness, and ability to capture real-time sleep data over longer periods.

Demographics and Surgical History: Demographics were collected from the electronic health record and confirmed by participants using surveys (e.g., age, gender, race, etc.). Data on surgery type and location were also collected from health records.

### 2.4 Statistical analysis

Statistical analyses were conducted using R (RStudio 2023.12.1). Means and standard deviations (±) were calculated as descriptive statistics for all continuous variables. Linear regression models assessed the relationship between Sleep Hygiene Index on the outcomes, which included PROMIS Pain Interference, as well as actigraphy derived Total Sleep Time and Sleep Efficiency. Given the potential confounding introduced by age and sex on outcomes, separate analyses fitting these factors into the models were examined. Statistical significance was set at *p* < .05.

## 3 Results

For this analysis, 13 participants were excluded due to missing actigraphy data and 1 participant was excluded due to missing survey data resulting in a total of 30 participants. The average age of the sample was 61.7 (±11.7) (Table 1). The majority of participants identified as female (73.7%), white (76.7%), and underwent an arthroplasty (60.0%) surgery. The average preoperative Sleep Hygiene Index score was 10.9 (±6.7) and PROMIS Pain Interference score was 63.7 (±9.6). The average actigraphy wear time was 3.5 days before surgery (±1.5 standard deviation [SD]). Data gathered from the actigraphy devices indicated that participants had an average Total Sleep Time of 363.0 (±154.0) minutes, and an average Sleep Efficiency score of 92.0% (±3.8). Participants in this sample had an average of 10.06 (±5.42) awakenings per sleep period, with an average wakefulness after sleep onset of 26.94 (±15.78) minutes. The average length of awakenings was 2.60 (±0.89) minutes, and participants had an average sleep fragmentation index of 4.84 (±10.16).

**Table 1:** Sample characteristics (*n* = 30).

Characteristics	Value
Age, mean (SD)	61.67 (11.74)
Sex, (%)	
Female	22 (73.3%)
Male	8 (26.7%)
Race, (%)	
White	23 (76.7%)
Black	7 (23.3%)
Type of surgery, (%)	
Arthroscopy	6 (20.0%)
Arthroplasty	18 (60.0%)
Other	6 (20.0%)
Surgical site, (%)	
Shoulder	26 (86.7%)
Elbow or other upper extremity	4 (30.3%)
Sleep hygiene index, mean (SD)	10.87 (6.71)
PROMIS pain interference, mean (SD)	63.73 (9.59)
Total sleep time, mean (SD)	362.97 (154.02)
Sleep Efficiency, mean (SD)	91.98 (3.72)
Wakefulness after sleep onset, mean (SD)	26.94 (15.78)
Number of awakenings per night, mean (SD)	10.06 (5.42)
Length of awakenings, in minutes, mean (SD)	2.60 (0.89)
Sleep fragmentation index, mean (SD)	4.84 (10.16)
Duration of actigraphy wear in days preoperatively, mean (SD)	3.5 (1.47)

**Figure 1:**
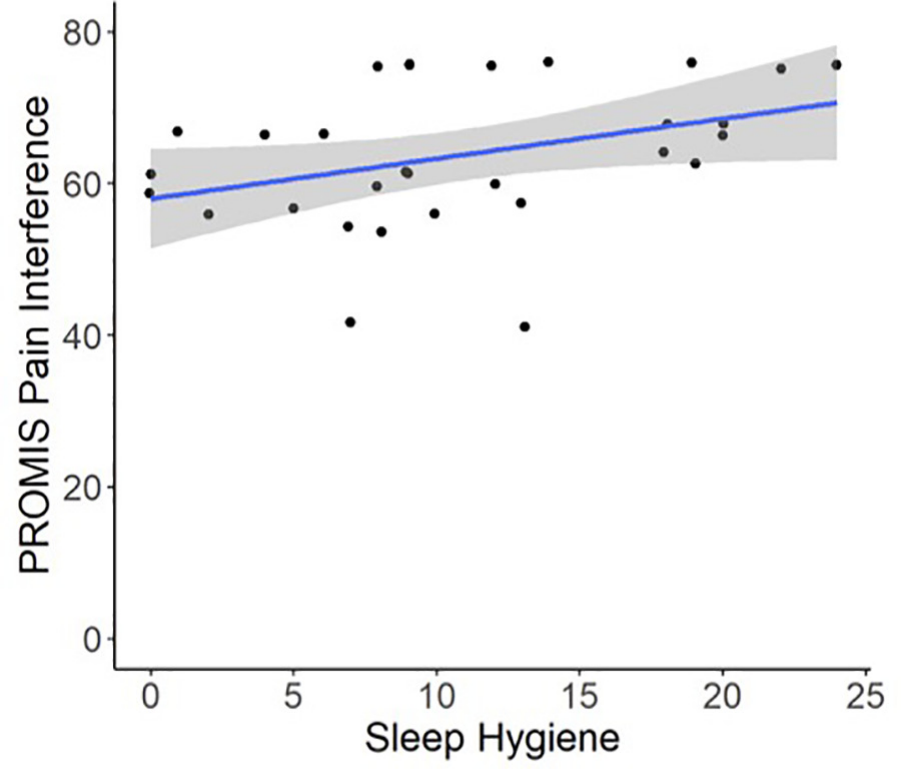
Regression illustrating each 1-point increase in Sleep Hygiene Index scores, indicating worse sleep environment or routine, was associated with a 0.53-point increase in PROMIS Pain Interference Scores preoperatively.

**Figure 2:**
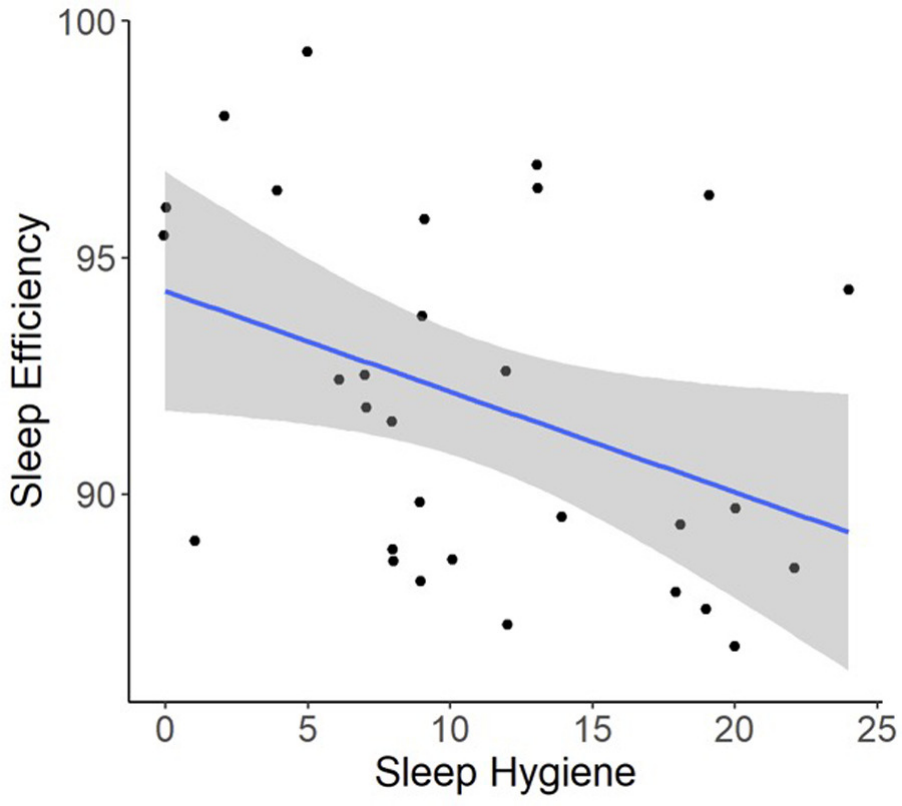
Regression illustrating each 1-point increase in Sleep Hygiene Index scores, was associated with a decrease in sleep efficiency over the preoperative period.

**Figure 3:**
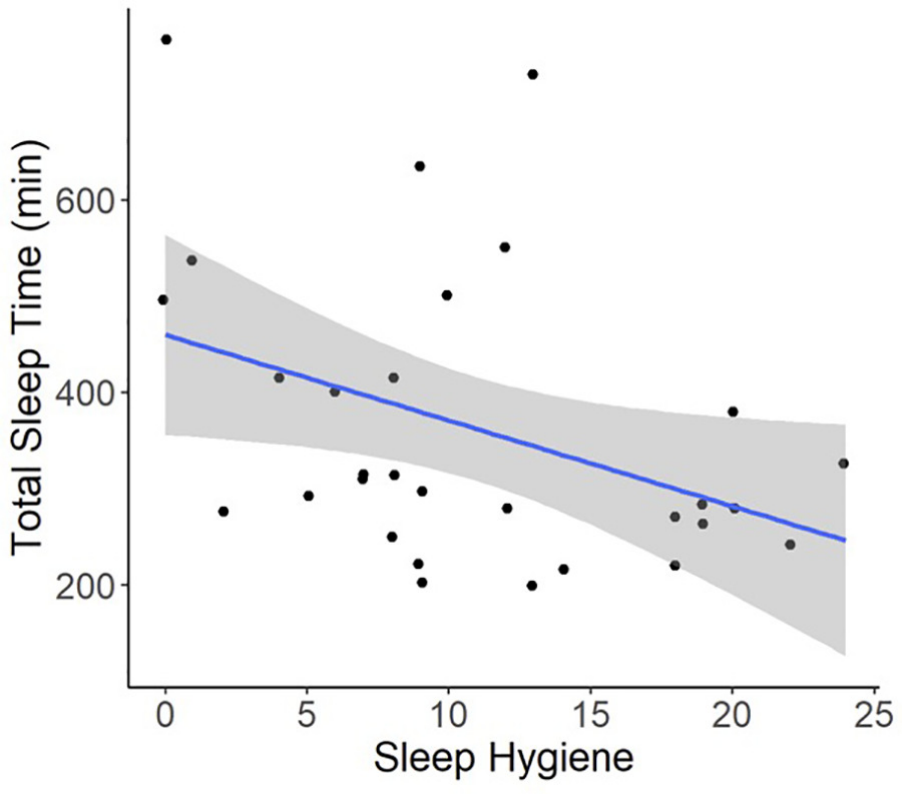
Regression illustrating each 1-point increase in Sleep Hygiene Index scores, indicating worse sleep environment or routine, was associated with an 8.9-minute decline in total sleep time over the preoperative period.

Regression models indicated that individuals with worse pre-operative Sleep Hygiene Index scores also experienced worse patient-reported pain outcomes and worse sleep metrics (Table 2). For example, models showed each 1-point increase in Sleep Hygiene Index scores corresponded to over a half point increase in PROMIS Pain Interference scores (*β* = 0.53; 95% CI: 0.14, 1.04; *p* = 0.044) (Figure 1). In addition, participants with worse Sleep Hygiene Index scores had lower Sleep Efficiency (*β* =  −0.21; 95% CI: −0.41, −0.01; *p* = 0.037) (Figure 2) and experienced fewer minutes in their Total Sleep Time (*β* = −8.91; 95% CI: −17.10, −0.72; *p* = 0.034) (Figure 3). These findings indicate that sleep environment, bedtime routine, and sleep behaviors may be linked to participants' pain and sleep presentations prior to undergoing upper extremity orthopaedic procedures. Neither sex nor age were significantly associated with any outcomes when examined as potential confounders (Supplementary Table S1, Supplementary Table S2).

**Table 2:** Regressions for sleep hygiene.

	Outcomes								
	Pre-surgical pain interference			Pre-surgical sleep efficiency			Pre-surgical total sleep time		
	*β*	95% CI	*P*	*β*	95% CI	*P*	*β*	95% CI	*P*
Intercept	57.98	51.45, 64.52	<.0001	94.29	91.76, 96.82	<.0001	459.76	355.63, 563.88	<.0001
Sleep hygiene index score	0.53	0.14, 1.04	.0443	−0.213	−0.41, −0.014	.0371	−8.91	−17.10, −0.72	.0341

## 4 Discussion

This analysis identified that higher pre-operative sleep hygiene scores were linked to patients experiencing greater pain interference and worsening sleep quality in the days prior to undergoing upper extremity orthopaedic surgery. Specifically, participants with higher, or worse, Sleep Hygiene Index scores pre-operatively were observed to have a higher PROMIS Pain Interference, fewer minutes of Total Sleep Time, and worse Sleep Efficiency. The results of this analysis posit that better sleep hygiene practices may be associated with gains in sleep duration, quality of sleep, and pain levels prior to surgery. Targeted strategies aimed at improving patients' sleep environments could aid in improving preoperative pain interference. For example, based on the modelling in this analysis an improvement of 6-points on the Sleep Hygiene Index would correspond to a minimal clinically important difference of over 3.3-points on the PROMIS Pain Interference measure (26). In the context of this sample, where average PROMIS Pain Interference scores were a standard deviation higher than population norms, addressing factors that can drive improvements in pain even prior to surgery may help mitigate a poor postoperative recovery (27, 28). Furthermore, even modest improvements in participants' preoperative sleep environment may help improve sleep architecture and duration. This research builds upon previous investigations studying sleep quality among patient populations undergoing surgery.

Poor sleep quality before surgery can lead to protracted recovery and place patients at higher risk of experiencing postoperative pain complications, yet there is a shortage of studies that incorporate patient-reported outcomes and actigraphy aimed at examining modifiable factors that may improve sleep in the preoperative period. Sleep quality and deprivation are known drivers of increased pain sensitivity and severity (8). This is of particular concern among patients undergoing surgical procedures. Meta-analyses and reviews have underscored the contributions of poor preoperative sleep on worsening postoperative pain (3, 29). However, basing sleep quality off cross-sectional survey-based assessments may not capture changes in daily sleep patterns as is possible with actigraphy. This study's incorporation of both patient-reported outcomes and objective actigraphy measures to examine preoperative presentations improves upon previous investigations in orthopaedic settings. Reviews indicate there is limited research utilizing objective measures of sleep to determine sleep architecture in surgical populations (3). Ongoing assessments, incorporating multiple assessment approaches, can guide future intervention development aimed at improving sleep quality. Studies have documented sleep disruptions in patients after undergoing surgical procedures, including cancer-related, arthroscopic, and cardiac surgeries, with less attention on upper extremity patient populations (30–32). Bolstering sleep quality and duration is of particular interest in samples similar to the present study, given the average preoperative nightly sleep time falls short of national recommendations for older adults (33). The average sleep time and efficiency seen in this sample reflect observations in other orthopaedic studies conducted with similar sample sizes preoperatively (34). Uniquely, our study not only captured sleep quality and pain presentation preoperatively but also assessed sleep behaviors.

While much attention has focused on postoperative sleep and its relationship to postoperative recovery and pain interference few investigations consider participants’ sleep hygiene, a potentially modifiable factor to address prior to surgery. Experts indicate there is a need to screen patients' sleep presentations preoperatively to inform care referrals for patients at risk for experiencing poor postoperative outcomes due to sleep (3). Assessing sleep hygiene preoperatively can help improve patient outcomes, guide personalized interventions, and potentially uncover new therapeutic strategies for optimizing both sleep and recovery. Targeted interventions aimed at improving sleep routines and environments have been found to improve sleep quality and duration in a number of patient populations and across in-patient settings (35–37). However, less research has examined patients' sleep hygiene prior to orthopaedic surgery on an upper extremity. Sleep routines are routinely disrupted following upper extremity surgery as many patients adjust to wearing a sling or sleeping in new positions, or even recliners (38). As such, improving preoperative sleep hygiene may aid in promoting a restful sleep environment postoperatively. Establishing good sleep hygiene preoperatively may facilitate high quality restorative sleep postoperatively, a period for many patients when sleep quality suffers after orthopaedic surgery (39). Continued research on changes in sleep behaviours prior to and after surgery among patients undergoing upper extremity orthopaedic surgery is needed.

### 4.1 Limitations

The observational nature of this work has inherent limitations. Modelling was able to discern associative relationships between sleep hygiene and outcomes, not causal. While sufficiently powered and similar in size to other investigations utilizing wearable devices to assess sleep architecture, our modest sample size may limit generalizability (40). Additionally, the stringent inclusion criteria, which helped better isolate presentations of joint specific pain, may not reflect the larger patient population undergoing upper extremity surgery. Although patients with diagnosed sleep related conditions were excluded, patients were not evaluated by a sleep professional to rule out an undiagnosed sleep disorder. Wear time compliance was low over the study period, evident by the average wear time of 3.5 days. However, research indicates that at least two nights of recorded sleep data are recommended when assessing actigraphy measures (41). In the context of this study 31 (77.5%) of the 44 participants consented and enrolled met the 2-day criterion and were eligible to included in the analysis. While this study did not examine postoperative outcomes, the use of repeated daily actigraphy metrics improves upon cross sectional studies focused on preoperative sleep and pain presentations. Despite these limitations, the use of validated PROMIS measures, paired with repeated daily objective actigraphy metrics, improves upon previous research examining preoperative sleep that are cross-sectional or depend upon registry-based data sources alone.

## 5 Conclusions

This analysis indicates that worsening sleep hygiene is associated with both increased pain interference and poorer sleep quality among patients about to undergo orthopaedic surgery on their upper extremity. These findings highlight the need to assess and educate patients on proper sleep hygiene prior to surgery in an effort to help foster high-quality restorative sleep that promotes postoperative recovery. This study is among the first to examine the possible contributions of sleep hygiene, a modifiable factor, on both patient-reported outcomes and objective measures of sleep over a prolonged period among patients undergoing orthopaedic surgery in an ambulatory setting. The outcomes of this study can inform future research aimed at guiding the development of patient centered interventions aimed at minimizing sleep disturbances preoperatively and throughout the postoperative period by promoting sleep hygiene. Future longitudinal research assessing the efficacy and sustainability of sleep hygiene interventions in enhancing surgical recovery outcomes is warranted. Collectively, findings from this study indicate that participants presenting with worse sleep hygiene report worse preoperative outcomes. Continued monitoring of sleep presentations is warranted to ensure timely implementation of interventions to promote high quality sleep to potentially optimize postoperative recovery.

## Data Availability

The raw data supporting the conclusions of this article will be made available upon reasonable request with IRB approval and data use agreement.
