# Supplementary material for: Sleep hygiene linked to patient-reported outcomes & objective sleep measures prior to upper extremity orthopaedic surgery
Source: Front Pain Res (Lausanne). 2025 Jun 11;6:1589748. doi: 10.3389/fpain.2025.1589748 (PMC12187727; doi:10.3389/fpain.2025.1589748)
Supplement: Supplementary file 1 [file Table1.docx]

Supplemental Table 1: Regressions for Sleep Hygiene accounting for Sex

|  | PROMIS Pain Interference | | | Pre-Surgical Sleep Efficiency | | | Pre-Surgical Total Sleep Time | | |
| --- | --- | --- | --- | --- | --- | --- | --- | --- | --- |
|  | β | 95% CI | P | β | 95% CI | P | β | 95% CI | P |
| Intercept | 53.39 | 45.09,61.69 | <.001 | 94.17 | 90.78,97.56 | <.001 | 462.63 | 323.15, 602.12 | <.001 |
| Sleep Hygiene Index Score | 0.52 | .02,1.02 | .039 | -0.21 | -0.42,-0.01 | 0.406 | -8.90 | -17.26, -0.55 | .038 |
| Sex | 6.32 | -1.10,13.74 | .912 | 0.17 | -2.86,3.19 | .912 | -3.96 | -128.59, 120.67 | .950 |

Supplemental Table 2: Regressions for Sleep Hygiene accounting for Age

|  | PROMIS Pain Interference | | | Pre-Surgical Sleep Efficiency | | | Pre-Surgical Total Sleep Time | | |
| --- | --- | --- | --- | --- | --- | --- | --- | --- | --- |
|  | β | 95% CI | P | β | 95% CI | P | β | 95% CI | P |
| Intercept | 65.37 | 43.46,87.28 | <.001 | 87.79 | 79.66,95.91 | <.001 | 682.97 | 342.85,1023.1 | <.001 |
| Sleep Hygiene Index Score | 0.47 | -0.07,1.02 | .086 | -0.16 | -.036,0.04 | .109 | -10.62 | -19.05,-2.18 | .0160 |
| Age | -0.11 | -0.42,0.20 | .475 | 0.09 | -0.02,0.21 | .096 | -3.32 | -8.14,1.50 | 0.160 |
